# School screening and point prevalence of adolescent idiopathic scoliosis in 4000 Norwegian children aged 12 years

**DOI:** 10.1186/1748-7161-6-23

**Published:** 2011-10-24

**Authors:** Raphael D Adobor, Silje Rimeslatten, Harald Steen, Jens Ivar Brox

**Affiliations:** 1 Department of Orthopaedic Surgery, Section for Spine Surgery. Oslo University Hospital- Rikshospitalet. Sognsvannsveien 20, Oslo, 0372, Norway; 2 Danmarksveien 59, Svarstad, 3275, Norway; 3 Department of Orthopaedic Surgery, Biomechanics Lab. Oslo University Hospital-Rikshospitalet. Sognsvannsveien 20, Oslo, 0372, Norway

## Abstract

**Background:**

School screening for adolescent idiopathic scoliosis (AIS) is discussed. The aim of the present study was to describe the point prevalence of AIS and to evaluate the effectiveness of school screening in 12-year- old children.

**Methods:**

Community nurses and physical therapists in the Southern Health region of Norway including about 12000 school children aged 12 years were invited to participate. All participating community nurses and physical therapists fulfilled an educational course to improve their knowledge about AIS and learn the screening procedure including the Adam Forward Bending Test and measurement of gibbus using a scoliometer.

**Results:**

Sub-regions including 4000 school children participated. The prevalence of idiopathic scoliosis defined as a positive Adam Forward Bending Test, gibbus > 7° and primary major curve on radiographs > 10°, was 0.55%. Five children (0.13%) had a major curve > 20°. Bracing was not indicated in any child; all children were post menarche; four had Risser sign of 4, and one with Risser 1 did not have curve progression > 5° at later follow-up. In one of these 5 children however, the major curve progressed to 45° within 7 months after screening and the girl was operated.

**Conclusion:**

The point prevalence of AIS in 12- year old children is in agreement or slightly lower than previous studies. The screening model employed demonstrates acceptable sensitivity and specificity and low referral rates. Screening at the age of 12 years only was not effective for detecting patients with indication for brace treatment.

## Background

There is a wide variation in the reported prevalence of adolescent idiopathic scoliosis (AIS). One study suggests that about 2.0% of adolescent children are found with screening to have scoliosis with a Cobb angle of > 10°, about 0.5% > 20°, and only 0.1% > 40°[1]. A review of twenty peer-reviewed papers shows a wide range variation of AIS prevalence in different countries with higher prevalence rates in the northern geographic latitudes and lower prevalence rates as the latitude is approaching the equator.(Finland 12%, Singapore 0.9%.) [2]. The prevalence of scoliosis > 20° in Scandinavia is reported to be 1.1% for girls and 0.1% for boys in another study [3]. Point prevalence is a measure of the proportion of people in a population who has a disease or condition at a particular time or at a particular age, by example one-month prevalence of back pain or prevalence of scoliosis at school screening in 12 year-old children. Point prevalence rates of AIS have been shown to increase with age; from 0.1% in the age-group of six to eight years, to 0.3% in the age-group of nine to eleven years, and 1.2% in the age-group of twelve to fourteen years [4].

Screening for scoliosis has been practiced worldwide for many years and has provided valuable knowledge about prevalence, aetiology and the natural history of idiopathic scoliosis. School screening for scoliosis beyond its scope of early identification of AIS has contributed to the field of research for aetiology of idiopathic scoliosis. Numerous factors that are implicated in the aetiology of AIS including biological factors such as menarche, lateralisation of the brain, handedness, the thoracic cage, the intervertebral disc, and the role of melatonin have been studied in children referred from school screening programmes [5]. Early diagnosis allows for bracing that is reported to be effective by numerous outcome studies [6-8], although the evidence is weak according to a recent Cochrane report [9]. In 1995, The United States Preventive Services Task Force advised against scoliosis screening [10,11]. Later publications suggest that they might not fully recognise data answering some of their objectives at the time of their recommendation [6]. In recent years, The Scoliosis Research Society and the American Academy of Orthopaedic surgeons, the Paediatric Orthopaedic Society of North America, and the American Academy of Paediatrics have endorsed scoliosis screening while The Canadian Task Force on the Periodic Health examination, the British Orthopaedic Association, and the British Scoliosis Society do not recommend screening [12,13].

The effectiveness of scoliosis screening is therefore still under debate. Objections to scoliosis screening are largely based on the low prevalence rate of clinically significant scoliosis, the inverse relationship of sensitivity and specificity in the screening process, high rates of false-positive cases, high inter-observer variations and the costs involved mainly because of over-referrals [14,15]. The challenge in scoliosis screening programmes therefore is to decrease the sensitivity to an acceptable rate of false positive results and to increase specificity in order to reduce over-referrals thereby reducing costs for the patients and society.

Based on the recommendations from 1995, routine scoliosis school screening programmes have been discontinued in many Western countries including Norway in the last 10-15 years. In Scandinavian countries, Sweden has conducted school screening for many years and has an ongoing scoliosis screening programme [7]. In Denmark, there have been attempts to perform school screening, but no specific scoliosis screening programmes have been successfully implemented (personal communication with Andersen, M.O.)

The effects of the discontinuation of scoliosis school screening programmes in Norway have not been thoroughly evaluated. However, a preliminary review of the referral records at the Oslo University Hospital suggests that fewer children with AIS are being detected early enough to benefit from brace treatment (unpublished data).

In Canada, school scoliosis screening has been discontinued since 1979 when the Canadian Task Force on the Periodic Health Examination did not recommend screening. The impact of this discontinuation has recently been examined. This report shows that, in subjects with confirmed AIS, 32% were classified as too late referrals with regards to brace treatment. The discontinuation of the school screening programmes was therefore followed by a suboptimal appropriateness of referrals for bracing [16].

The optimal age for scoliosis screening is still under debate. School screening has generally been performed between the ages of 10 to 14 years in conjunction with a school health examination [10,17]. The Scoliosis Research Society has recommended annual screening of all children aged 10-14 years. The American Academy of Orthopaedic Surgeons has recommended screening girls at 11 and 13 years and screening boys at age 13 or 14 years. The American Academy of Paediatrics has recommended annual scoliosis screening with the forward bending test at routine health supervision visits.

The combination of the Adam forward bending test and the scoliometer measurement of the angle of trunk rotation (ATR) has been shown to be the simplest, quickest, most reliable, and least expensive objective measure of trunk deformity [18]. It has been recommended that an inclination above 7° or ATR > 1 cm is a positive screening sign and should be followed-up with an X-ray for further evaluation of the curve [19].

The present study was designed to evaluate the point prevalence, and the effectiveness of school screening of AIS in a Norwegian population of 12000 children aged 12 years.

## Methods

### Study design

Screening of idiopathic scoliosis was performed in conjunction with the ordinary school health examination and vaccine programme in 12 year- old children in the Health Region South of Norway which has a population of about 12000 children at this age.

### Sample selection

There is a similar distribution of girls and boys in the population at target and in the population screened. The sex distribution in the group with positive screening and in those with scoliosis at x-ray examination, are reported in the results section.

### Preparation for school screening

Public health/community nurses and physical therapists in the study region were engaged as screeners. They were invited to a one-day intensive course at the Oslo University Hospital, Rikshospitalet to improve their knowledge about AIS. Additional courses were arranged at the various county centres for those who were not able to attend. Participants were taught about scoliosis and the screening procedure of Adam Forward Bending Test and measurement of the angle of inclination using the scoliometer. In addition, a scoliosis screening manual was provided to all participants and follow-up teachings were provided as needed.

### Screening technique

The screening procedure combined the standing visual inspection of the back, the Adam Forward Bending Test and the scoliometer (OSI-scoliometer Orthopaedic Systems Inc, Hayward, California, USA) measurement of angle of trunk rotation (ATR). Seven degrees of ATR was chosen as cut-off point for referral to radiography [20-22].

### Referral criteria and treatment

Radiographic results from screening at local hospitals were mailed to the Department of Orthopaedics at Oslo University Hospital-Rikshospitalet. A Cobb angle > 10° on standing radiographs were classified as AIS according to the criteria proposed by the Scoliosis Research Society [23].

Scoliosis between 10° to 20° were referred to a new radiographic exposure within 6 months and Cobb angles > 20° were referred for physical examination and new standing X-rays including crista crest exposure for Risser sign grading.

### Statistical analysis

We estimated that the population of boys and girls were equal in the examined population and calculated the point prevalence of AIS. We also estimated the point prevalence of scoliosis > 10° from the reported prevalence in two previous epidemiological studies [24]. Based on these studies, we used 0.8% as the point prevalence rate of scoliosis in the study population to estimate the sensitivity and specificity of the screening procedure used.

Sensitivity is a measure of a test's ability to identify positive results. It is calculated from the ratio of true positives to combined true positive and false negatives. Specificity measures a test's ability to identify negative results. Specificity is calculated from the ratio of true negatives to combined true negatives and false positives.

Additional parameters determining reliability of the screening procedure such as positive predictive value, (PPV), negative predictive values, (NPV) and likehood ratios (LR+, and LR-) were also calculated [25].

Positive predictive value (PPV) is the proportion of patients with positive test results who are correctly diagnosed, and negative predictive value (NPV) is the proportion of patients with negative test results who are correctly diagnosed.

Likelihood ratios are normally used for assessing the value of performing a diagnostic test. They use the sensitivity and specificity of the test to determine whether a test result usefully changes the probability that a condition exists. Two versions of the likelihood ratio exist, one for positive and one for negative results.

## Results

Of the 12000 twelve year- old children living in different regions of Health Region South, we were able to screen only sub-regions including 4000 twelve year old school children. Since screening has been discontinued in Norway, the Directory of Healthy in Norway was not willing to support the programme with a recommendation. Many community nurses and physical therapists were not willing to conduct a task that was not recommended and participation in the programme was therefore lower than expected.

Sixty pupils were found positive on both standing, forward bending test and scoliometer measurements > 7°. There were 39 (65%) girls and 21 (35%) boys. Twenty-two were confirmed with scoliosis on standing radiographs, 16 (73%) girls and 6(27%) boys. Thirty-eight of which 23 (60%) girls and 15 (40%) boys had normal spine curvatures on X- ray examination (false positive). These were followed up until maturity and none progressed to > 25°. The referral rate to radiography from screening was 1.5% and point prevalence of confirmed scoliosis was 0.55%.

Five girls with clinical and radiographic significant scoliosis (> 20°) were discovered with screening, (Table 1). All were post menarche. Four had Risser sign of 4 and were more than 1 year post menarche. Brace treatment was therefore not indicated in any of them. One girl had Risser 1, but was more than one year post menarche; the major curve did not progress > 5° within 6 months, and brace treatment was therefore not indicated. Scoliosis in four of the girls did not progress > 5°during long-term follow-up. In one of them the scoliosis progressed from 37°to 45° within 7 months after screening and she was operated. The point prevalence of curves > 20° was 0.13% in girls and 0.0% in boys.

**Table 1:** Follow-up of children with scoliosis > 20° at screening.

Patient	Age at screening	Major curve at screening	Risser sign	Post Menarche	Major curve at follow up	Treatment status
1	13	37° thoracic 32° lumbar	4	12 months	45° thoracic 43° lumbar	Posterior fusion
2	12	27° thoracic 16° lumbar	1	16 months	27° thoracic 21° lumbar	Observation
3	12	16° thoracic 24° lumbar	4	16 months	19° thoracic 24° lumbar	Observation
4	12	30° thoracolumbar	4	2 months	30° thoracolumbar	Observation
5	12	29° thoracic	4	24 months	29° thoracic	Observation

Eleven girls and 6 boys had curves between 10° and 20° and they were observed for further progression until maturity. None of them progressed to > 25°.

With an estimated point prevalence rate of scoliosis of 0.8% in 12 year- old children, the sensitivity was calculated to be 69%, the specificity was 99%, positive predictive value was 37%, and the negative predictive value was 99%. The positive likelihood ratio (LR+) was 46 and the negative likelihood ratio (LR-) was 0.55 (Table 2).

**Table 2:** Contingency table showing the calculations of parameters of reliability of the screening test.

Population 4000		
	Children with Scoliosis	Children without scoliosis
Positive Screening	True Positive (**TP**) 22	False Positive(**FP**) 38

Negative Screening	False Negative(**FN**) 10	True Negative(**TN**) 3962

Sensitivity = TP/(TP+FN) 22/32 = 0.69

Specificity = TN/(FP+TN) 3962/4000 = 0.99

PPV (positive predictive value) = TP/(TP+FP) 22/60 = 0.37

NPV (negative predictive value) = TN/(FN+TN) 3968/3962 = 0.99

LR+ (positive likelihood ratio) = Sensitivity/(1- specificity) 0.69/0.01 = 69

LR-(negative likelihood ratio) = 1-sensitivity/(specificity) 0.31/0.99 = 0.31

## Discussion

There is a wide variation in the reported prevalence of AIS. Most studies have reported that about 2.0% of adolescent children are found on screening to have scoliosis with a Cobb angle > 10°[1]. Point-prevalence is the prevalence based on a single examination of everyone in the population at one point in time which will probably underestimate the true prevalence of AIS.

The point prevalence applied in the present study was based on examination in 12 year-old children and because there is a large variety of the start of puberty and scoliosis, the study could underestimate the true prevalence of AIS. The present study has shown a point prevalence of 0.55% for scoliosis.

The observed point prevalence rate in 12- year- old children in the current study corresponds well with previous studies reporting the age-specific prevalence for 9-11 and 12-14 years.

The prevalence rates in previous studies however are not easily comparable because they do not exclusively refer to AIS and different age groups are usually included. The prevalence rate could be different if various Cobb angles of > 5°, 10°or 20°were used and if non-structural scoliosis were included. The point prevalence of AIS in 12-year- old children in the present study was 0.40% in girls and 0.15% in boys which reflects the later onset of puberty in boys.

### Optimal age of screening

The optimal age for scoliosis screening is still under debate. School screening has generally been performed between the ages of 10 to 14 years in conjunction with a school health examination. Ideally screening should be performed in girls before the onset of menses and 1-2 years later for boys. The challenge in screening is to detect clinically significant curves in immature children which have the potential of progression.

The girls with a significant scoliosis curve of > 20° in the present study were all judged to be too mature for brace treatment. This suggests that screening should have been performed one year earlier. The prevalence rate of 0.55% in the present study as compared with 1.1% in girls in previous studies most likely reflects the wide range of onset of puberty [3], and the fact that only 12-year-old children were examined. Age at menarche is considered a reliable prognostic factor for AIS and varies in different geographic latitudes. AIS prevalence has also been reported to be different in various latitudes, with higher values in northern countries. The point prevalence of AIS in 12 year-children in the present study does not compare well with the reported 12% prevalence of AIS in Finland [2], but rather with the 1.1% rate found in another report about of AIS prevalence in the Scandinavian countries [3].

Radiological skeletal maturity was evaluated by Risser sign only in the present study, while bone age assessment from the left hand (Greulich & Pyle, 1959) or elbow (Sauvegrain) is most used world-wide [26]. In one of the girls with Risser sign of 4, and 1 year post-menarche at screening, her major curve progressed from 37°to 45° within 7 months. Additional assessment of skeletal age at screening might have provided important supplemental information.

### Rationale behind scoliosis screening

The prediction of scoliosis progression depends largely on skeletal maturity and curve magnitude. Larger curves in immature patients have higher risks of progression than smaller curves in more mature patients. The rationale behind screening is therefore to enable early detection of curves > 20° in immature patients that permits initiation of bracing which may halt progression, or allow surgery at appropriate time and avoid the complications of surgery of advanced scoliosis.

### Effectiveness of scoliosis screening

Direct evidence of the effectiveness of scoliosis screening would require controlled prospective studies demonstrating that persons who receive screening have better outcomes than those who are not screened. Documentation is limited, but few studies including a recent study from the Netherlands, have demonstrated that scoliosis cases detected through screening had lower chances of having surgery than otherwise detected patients [27,28]. There are some studies reporting that patients with scoliosis detected by screening are younger than referred cases, have smaller curve size, and reduced risk to progress to > 45°, and thereby having surgery. On the other hand, the number of referrals to local scoliosis clinics is increased by screening [29-32].

The current study was designed to screen 12000, twelve year old children but we were able to screen only 4000. Since screening has been discontinued in Norway, the health authorities did not support the study with a recommendation that could have boosted participation in the study. The study did neither include sufficient school children nor a follow up to evaluate whether those children screened have a better outcome than those not screened.

### Accuracy of screening tests

The sensitivity and specificity of scoliosis screening depends largely on the skills of the examiner and the magnitude of the scoliosis being sought. The use of scoliometer has been shown to increase the sensitivity and the specificity in detecting a Cobb angle of > 20° [33]. A scoliometer reading of 5° has been shown to have a sensitivity of 100%, and 47% specificity for identification of scoliosis, whereas a scoliometer reading of 7° increases the specificity to 86% but decreases the sensitivity to 83% [34]. In the present study, using a scoliometer reading of 7°, the sensitivity was 69% and the specificity was 99% in detecting AIS in the study population.

The positive predictive value of visual inspection and the forward-bending test varies with the degree of curvature by which a "true positive" is defined, the prevalence of scoliosis in the screened population, and the skills of the examiners [35,36]. The magnitude of PPV is thus inversely related to the degree of curvature used to define scoliosis since the prevalence of small curves is greater than large curves. In a study from Australia, the PPV was 78% for curves > 5° in a population with an estimated prevalence of 3% [37]. In another study, the PPV was 54% for curves > 10° with a predicted prevalence of 2%, and 24% for curves > 20° with an estimated prevalence rate of 0.3% [36]. A meta-analysis of the clinical effectiveness of school scoliosis screening citing 34 studies reported that the pooled PPV for curves > 10°, curves > 20°, and treatment were 28.0%, 5.6% and 2.6%, respectively [38]. In the present study, the PPV was found to be 37% applying the accepted > 10° definition of scoliosis and an estimated point prevalence of 0.8% in 12 year- old children.

A likelihood ratio > 1 indicates that a test result is associated with a disease and a likelihood ratio < 1 indicates that a test result is associated with the absence of a disease. It is when the positive likelihood ratio is > 5 or the negative likelihood ratio is < 0.2 that likelihood ratios can be applied to the pre-test probability of a patient having a specific diagnosis. In this present study, the positive Likelihood ratio was calculated to be 69 and the negative likelihood ratio was 0.3. The screening model was sensitive enough to reduce the number of false positive results. However, since the number of abnormal radiographs of Cobb angle > 10° (true scoliosis) is not known in the study population, the true PPV cannot be known.

## Referral rates

Referral rates have been reported to be as high as 21% without the use of objective criteria, but reduced as much as 90% by the use of objective criteria [20,39,40]. A 3% referral rate has been predicted using 7° scoliometer reading, as compared to a referral rate of 12% using 5°scoliometer reading [20]. A Meta-analysis of the clinical effectiveness of school scoliosis screening citing 34 studies reported the pooled referral rate to radiography of 5% [38]. It is now widely agreed upon that referral rates should be in the range of 2% to 3% in school screening for scoliosis [20,39,41,42]. In the present screening study the referral rate was 1.5% based on a scoliometer reading of 7° which may reflect that screening was conducted on 12 -year- old children only.

In the present study, 38 children were falsely diagnosed as AIS (positive on screening but had normal spine on radiography). If screening was performed yearly nationwide, the total estimated number of children with negative radiographs (Cobb angle < 10°) in 60000 children of 12 years in the Norwegian population of 5 million inhabitants will be 570 which might be a concern for health authorities.

Screening for scoliosis using the scoliometer does not reveal scoliosis per se but detects thoracic deformity. The radiographic measured thoracic Cobb angle has been shown not to correlated to the rib-index (that is the surface deformity) in the younger group but only in the 14-18 years-old age group [43]. This lack of association of the surface asymmetry (hump) and radiological asymmetry (Cobb angle) in the younger group such as in our study, is creating the burden of false positive referrals and the negative attitude of several health decision boards to discontinue school screening programs in the various countries. Thus, it is not possible to reliably predict the degree of curvature from surface topography in the age group that are screened [6]. It has been reported that, in typical screening settings where the prevalence and positive predictive value are relatively low, for every curve > 10° detected, there are 1-5 false-positives; similarly, for every curve > 20° detected, there are 3-24 false-positives [11,43]. This number of false positive children on screening must be accepted if those with asymmetry, who might develop scoliosis should be detected.

The goal of screening is to detect those who will be at risk for developing scoliosis in the school- age population. In evaluation of the effectiveness of screening for scoliosis it should also be taken into account the knowledge gained and contribution it offers in clinical research of idiopathic scoliosis aetiology. The lack of a deeper insight on school screening issue, its value and negative impact of its discontinuation in some countries was the trigger for a recent decision of the Scoliosis Research Society (SRS) presidential line to create an International task Force for the better study of the school screening issue and creation of a "white paper" with recommendations based on recent knowledge on the topic [42].

### Potential adverse effects

It has been argued that screening could have psychological labelling effects to subjects, and increase exposure to radiographs. In the present study, attempts have been made to limit psychological labelling by providing adequate verbal and written information to children and parents before and after screening. We also tried to limit exposure to radiography by choosing a high cut-off ATR of 7° and providing adequate training for our screeners thereby reducing false positive findings which in turn reduce unnecessary exposure to radiography.

## Conclusion

The point prevalence of AIS in the present study is in agreement or slightly lower than results from earlier studies. The screening model employed demonstrates acceptable sensitivity and specificity, and low referral rates. The calculated likehood ratios are acceptable for a screening test. In the present study screening for scoliosis at the age of 12 years only was not effective for detecting patients with indication for brace treatment. Screening should probably be initiated one year earlier for girls and one year later for boys, or be conducted more than once. The costs and the use of health care resources and the radiation exposure should be considered when the screening criterion is chosen.

## Competing interests

None of the authors have received benefits for personal or professional use from a commercial party related directly or indirectly to the subject of this manuscript e.g., royalties, stocks, stock options, decision making positions.

## Authors' contributions

JIB and RDA designed the study. RDA and SR designed the screening programme and performed the educational courses for the screeners. RDA, JIB and SR were involved in the collection of the data for the manuscript. RDA examined all referred patients. RDA, JIB and HS were involved in the analysis and the interpretation of results, drafting and critical review of the manuscript. All authors have given final approval to the version to be published.
